# Establishing Functional Communication Responses and Mands: A Scoping Review of Teaching Procedures and Implications for Future Investigation

**DOI:** 10.3390/bs16020182

**Published:** 2026-01-27

**Authors:** Kyle W. Dawson, Amanda N. Zangrillo, Samantha J. Bryan, Rebecca J. Barall, Colin G. Wehr

**Affiliations:** 1 Munroe-Meyer Institute, University of Nebraska Medical Center, Omaha, NE 68106, USA; anzangrillo@cmh.edu (A.N.Z.); sam.bryan@unmc.edu (S.J.B.); bbarall@unmc.edu (R.J.B.); cwehr@unmc.edu (C.G.W.); 2 Developmental and Behavioral Pediatrics, Children’s Mercy Hospital, Kansas City, MO 64108, USA; 3 School of Medicine, University of Missouri-Kansas City, Kansas City, MO 64108, USA

**Keywords:** behavior reduction, functional communication response, functional communication training, mand training, verbal behavior

## Abstract

The functional communication response (FCR) shares fundamental properties with the mand, with both responses linking the relevant motivating operation and the reinforcer maintaining a response. The FCR differs from the mand in that the communication response has the expressed intention of replacing challenging behavior by providing an outlet to access the same functional reinforcer. Research describes the development of mand and FCR repertoires; however, no research to date elucidates differences and similarities in how these repertoires are established. A scoping review was selected to systematically map and compare instructional procedures across FCT and mand training studies. In this scoping review, we analyzed 98 peer-reviewed empirical studies published between 2014 and 2024 that taught FCR or mand repertoires, identified through searches of PsychINFO and ERIC using predefined inclusion and exclusion criteria. We (a) reviewed teaching strategies for developing mand and FCR repertoires, (b) analyzed unique and shared teaching features, and (c) identified implications for future research on teaching FCRs to replace challenging behaviors. Results showed that mand training studies more often targeted multiple responses to expand communication repertoires, whereas FCT studies typically focused on teaching a single response to rapidly suppress problem behavior. Additional distinctions included strategies for contriving motivating operations, prompting procedures, and communication topographies. These findings highlight important procedural divergences and suggest the need for integrated instructional approaches that promote generalization and functional use of communication.

## 1. Introduction

Communication is a fundamental aspect of human interaction that allows individuals to effectively express needs, wants, and information. One crucial type of communication is the request, or mand. B.F. Skinner introduced the concept of the mand in his 1957 book, Verbal Behavior (Skinner, 1957). He defined mands as a verbal operant uniquely characterized by its ability to specify its own reinforcer. Unlike other verbal operants that are evoked by external stimuli (e.g., tacts, intraverbals), mands are directly influenced by motivating operations (MOs; Michael, 1982), environmental events or internal conditions that momentarily alter the value of a reinforcer and the probability of behavior that has previously accessed it. A specific type of MO, known as an establishing operation (EO; Michael, 1993), increases the value of a specific reinforcer and evokes behavior that has historically produced that reinforcer. For example, water deprivation increases the value of water and evokes behavior historically associated with access to water, such as asking for a drink. This EO–response–reinforcer relationship is what defines the mand. The speaker’s response specifies the reinforcer, and the delivery of that reinforcer by the listener satisfies the EO, thereby completing the contingency. In contrast, abolishing operations (AOs; Michael, 1993) decrease the value of a reinforcer and abate related behavior, such as when satiation reduces the likelihood of requesting food.

Mands are critical responses that allow a speaker to get their wants and needs met through interaction with their verbal community. For instance, the dehydrated person who asks for water (reinforcer—access to water), a sleep-deprived parent who begs their newborn for just one hour of quiet to rest (reinforcer—escape from noise, access to sleep), or a curious toddler who inquires to a parent, “Why is the sky blue?” (reinforcer—access to information). These examples illustrate how EOs establish the value of particular reinforcers and evoke specific verbal behavior, reflecting the dynamic interplay between internal states and environmental contingencies. When individuals have an effective way to mand, they can access reinforcement in appropriate ways.

Recently published clinical tutorials dedicated to the instructional practices of mand training provide considerations for contriving, maintaining, and confirming EOs (Frampton et al., 2024a, 2024b). Frampton et al. (2024b) focused on procedures for contriving EOs through antecedent arrangements, such as an interrupted chain procedure or incidental teaching. The authors describe the teaching methods used with each strategy, methods to measure functional control and correspondence, and ethical considerations for each method. The second article (Frampton et al., 2024a) discussed how practitioners can use indicating responses as a method to suggest the presence of an EO for the specific reinforcers. The authors describe that, by ensuring the EO is present during teaching, clinicians can increase the probability that the verbal operant is a mand emitted to satisfy the current EO. Together, these articles provide a highly detailed roadmap for building mand repertoires with a high degree of instructional precision. Although focused on skill acquisition rather than behavior reduction, this level of specificity may hold value for informing instructional components of functional communication training (FCT) interventions that prioritize challenging behavior reduction in addition to acquisition of functional communication.

As language development occurs, an early learner may experience shifts in contingencies away from previously reinforced responses (e.g., crying), resulting in response variability and development of more complex language repertoires. When an individual experiences delays or deficits in acquiring language or navigating social interaction, challenging behaviors (e.g., aggression, self-injury) can emerge and persist, likely through unintentional or adventitious reinforcement. Challenging behavior concerns occur at an increased likelihood for youth diagnosed with developmental disabilities and autism spectrum disorder relative to same-aged peers (Kurtz et al., 2020). These individuals may experience difficulties developing language and social behavior and may contact reinforcement for challenging behavior. Thus, the environment might continue to favor challenging behavior responses due to potentially lower response effort and quicker access to reinforcement relative to more desirable communitive behaviors. Over time, this may result in the individual allocating responding towards challenging behavior, instead of the more desirable communication responses. Prioritizing language development early in the developmental window remains critical for early learners with and without disabilities.

Teaching mands is often a critical component of early intervention and skill acquisition programs because they are directly tied to the learner’s motivation, which increases the probability of acquisition (Sundberg & Michael, 2001). By systematically shaping and reinforcing mands, practitioners can build more functional and socially appropriate repertoires that allow individuals to effectively access reinforcement in a way that is understandable to their verbal community. When implemented early in a child’s development, mand training promotes independence and may serve as a preventative strategy, reducing the likelihood that individuals will resort to challenging behavior to get their needs met (Shillingsburg et al., 2013; Sundberg & Partington, 1998).

In cases where challenging behavior is already occurring at clinically concerning levels, a more targeted intervention may be necessary. FCT (Carr & Durand, 1985) involves identifying the specific reinforcer maintaining challenging behavior and teaching a functionally equivalent communicative response, known as a functional communication response (FCR), while often placing challenging behavior on extinction. While the FCR is a type of mand, as it is evoked by motivating operations and maintained by the specified reinforcers, mands and FCRs are not interchangeable; not all mands function as FCRs. FCRs are a specialized subset of mands that are explicitly taught within behavior reduction contexts to replace challenging behavior with an appropriate, functionally equivalent communicative response (Carr & Durand, 1985; Tiger et al., 2008). This distinction is critical, as the instructional goals and procedures used to establish FCRs often differ meaningfully from those used in traditional mand training. FCRs are typically taught to reduce challenging behavior within a formal behavior intervention plan, whereas mands are often taught to expand an individual’s ability to communicate effectively within their verbal community. The goal of challenging behavior reduction may influence how clinicians teach FCRs, such as placing increased emphasis on rapid suppression of the challenging behavior. As a result, the instructional strategies used to teach FCRs and mands may diverge based on the broader goals of the intervention, despite their shared goal of communicating wants and needs.

Tiger et al. (2008) provided a practitioner’s guide to implementing FCT, synthesizing over two decades of research following Carr and Durand’s (1985) seminal work. Their tutorial described key components of effective FCT implementation, including selecting appropriate response modalities (e.g., selecting a low response-effort replacement), prompting and reinforcement strategies (e.g., prompt delays, reinforcing FCRs while placing challenging behavior on extinction), and thinning reinforcement schedules to promote generalization and maintenance. The authors offered a broad and flexible framework for applying FCT across clinical contexts. However, while the article addresses the need to contrive EOs to create motivation, it provided limited detail on how these conditions should be arranged or embedded into programming. The lack of detail may reflect the authors’ intent to provide a broad implementation framework, rather than detailed guidance on arranging and delivering teaching trials.

The contrast between mand and FCT tutorials highlights a gap in our current understanding of how instructional procedures are described and applied across communication-based interventions. Tiger et al. (2008) offer a broad, practitioner-focused framework for implementing FCT, and Frampton et al. (2024a, 2024b) provide highly detailed guidance for teaching mands; however, it remains unclear to what extent FCT procedures are described with similar instructional precision across the literature. The additional goal of challenging behavior reduction may influence FCR teaching procedures. The ways in which FCT procedures converge or diverge from traditional mand training have not been systematically examined.

A scoping review methodology was selected because the primary aim of the present study was to identify, categorize, and compare instructional procedures used to establish FCR and mand repertoires across the applied behavior analysis literature. Unlike systematic reviews or meta-analyses, which typically focus on evaluating intervention effects or methodological quality, scoping reviews are well-suited for mapping how interventions are implemented, described, and differentiated across studies (Arksey & O’Malley, 2005; Peters et al., 2020). This approach allowed for a comprehensive examination of procedural features without excluding studies based on design characteristics or outcome magnitude.

Therefore, the goal of this study is to (a) review published teaching strategies for developing mand and FCR repertoires, (b) analyze unique and shared teaching features, and (c) provide considerations for future research when teaching an FCR to replace challenging behavior.

## 2. Methods

### 2.1. Search Strategy

**Figure 1:**
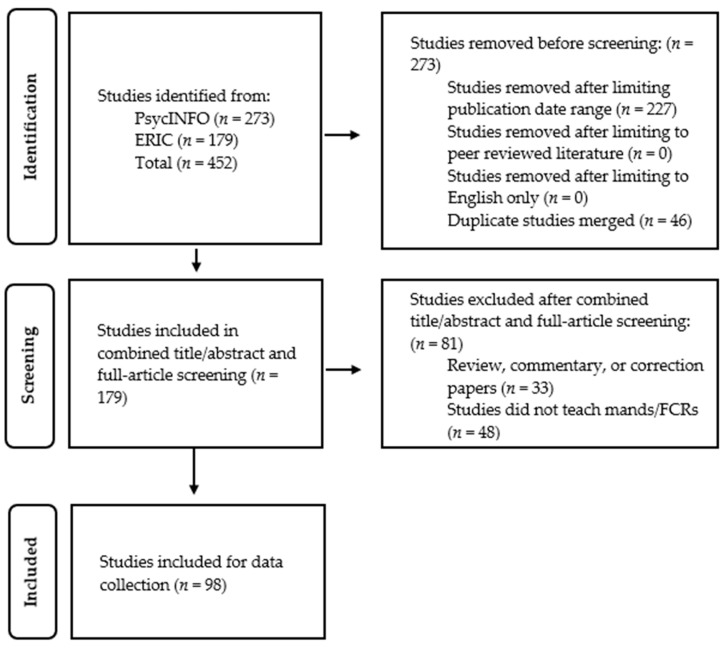
PRISMA Flowchart of Study Identification.

We conducted a literature search using procedures described in the Preferred Reporting Items for Systematic Reviews and Meta-Analysis (PRISMA) guidelines (Page et al., 2021). Figure 1 depicts a PRISMA flow diagram illustrating study identification, screening, eligibility, and final inclusion.

We searched PsycINFO and ERIC databases from 2014 to 2024 to identify empirical studies on FCT and mand training published in the last 10 years. We selected these dates to capture contemporary instructional practices in FCT and mand training. Limiting the review to the most recent 10 years allowed for a focus examination of current procedural trends while maintaining a manageable and interpretable scope consistent with the aims of a scoping review.

The search strategy included three sets of keywords combined using Boolean operators. The first set focused on intervention terms (e.g., “functional communication training,” “FCT,” “mands”). The second set included terms related to behavior analysis (e.g., “Applied Behavior Analysis,” “Verbal Behavior”). The third set specified six behavior-analytic journals (e.g., “Journal of Applied Behavior Analysis,” “The Analysis of Verbal Behavior”) used in this review. Journals were selected to represent widely read, high-impact outlets within applied behavior analysis. Impact factor was used as a general guide to identify influential journals rather than as a strict inclusion criterion. All keyword sets were linked using “AND” to refine the search.

This scoping review did not have a pre-registered protocol, which aligns with PRISMA-ScR guidance that protocol registration is recommended but not mandatory.

The PsycINFO search yielded 273 results, and the ERIC search produced 179 results, for a total of 452 articles. Table 1 depicts the Boolean search strategy.

### 2.2. Study Selection

After removing duplicate records, we applied inclusion and exclusion criteria to determine article eligibility. Studies were included if they: (a) were published between 2014 and 2024; (b) were empirical investigations; (c) appeared in one of the six preselected, peer-reviewed journals; (d) were written in English. We excluded review papers, theoretical discussions, dissertations, conference abstracts, and studies lacking an experimental control (e.g., case studies without experimental manipulations). Next, we conducted a combined title and abstract search and full-article screening to exclude any additional articles that did not meet the inclusion criteria. Data on the screening procedures can be found in the PRISMA flowchart (Figure 1) and in Section 3.

### 2.3. Data Extraction and Coding

The first author developed a coding system to systematically extract key variables from the selected studies. The coding system included an online codebook using Google Docs (Version current as of January 2026; Google LLC, Mountain View, USA) and two online surveys created using Google Forms (Version current as of January 2026; Google LLC, Mountain View, CA, USA). Survey responses were automatically compiled into a spreadsheet using Google Sheets (Version current as of January 2026; Google LLC, Mountain View, CA, USA) for analysis. The first survey gathered article information, such as title, first author, and overall study procedures. The first survey also included questions used to categorize interventions as either FCR or mand training. Instead of using the authors’ tact of the intervention either being FCT or mand training, the survey included a question to identify the type of intervention based on the presence or absence of a behavior reduction goal. The second survey gathered participant-specific information such as diagnoses, reported verbal skills, and any procedural modifications from the general procedures, if applicable. The first author also created a document that included coding instructions, website links, and operational definitions for all questions and responses. The coding instructions and operational definitions are included in Appendix A and Appendix B, respectively.

To calibrate the coding method and ensure agreement among data collectors, researchers independently coded one initial article and compared results. Data were collected in the online spreadsheet and analyzed using item-by-item agreement across all data collectors. In the first calibration, researchers independently scored data from the sample article. Any questions that did not have 100% agreement across all data collectors were discussed by the authors. The research team adjusted dependent variables, coding response categories, and operational definitions, as needed, until all data collectors agreed on the scoring information. A second calibration round was conducted with a second, different article, with researchers again reviewing and resolving any inconsistencies before coding the remaining studies. Following the second calibration, the first author finalized the coding instructions and operational definitions for the data collectors. Calibration data are not reported, as this process was completed as a group. Agreement scores for full data collection are provided later.

Coding procedures allowed for both mutually exclusive and non-mutually exclusive variables, depending on the construct being assessed. Variables such as presence or absence of behavior reduction and methods to contrive EOs were coded as mutually exclusive. In contrast, variables that allowed for multiple features to co-occur within a single application (e.g., function of behavior, prompting strategies) were coded as non-mutually exclusive, allowing multiple selections when applicable. This approach was used to accurately capture the complexity and variability of instructional properties described in the literature.

### 2.4. Critical Appraisal of Sources

In accordance with the objectives of this scoping review and standard recommendations for scoping methodologies, no formal critical appraisal of individual sources of evidence was conducted. The aim of this review was to identify and compare instructional strategies rather than evaluate the methodological quality or risk of bias included in the studies.

### 2.5. Interobserver Agreement

A second researcher independently coded 30.7% of the articles included in the title and abstract and full-article screening to assess interobserver agreement (IOA), resulting in IOA on both inclusion and exclusion of articles as well as for dependent variables assessed in the review. Agreement was calculated using point-by-point comparison, dividing the number of agreements by the total number of coding decisions and multiplying by 100. The average IOA across studies was 94.01% (range: 62.96–100%). Any discrepancies were resolved through discussion to maintain accuracy and reliability in data extraction.

To minimize bias, all included studies were selected using predefined inclusion and exclusion criteria, and data were extracted using a standardized codebook applied consistently across coders. Reviewers were trained to ensure objective interpretation of key concepts such as “mand” and “functional communication response,” and interobserver agreement procedures, as described above, were implemented to confirm consistency in coding. These safeguards were intended to promote transparency, objectivity, and ethical rigor in the review process.

## 3. Results

### 3.1. Articles

The search method yielded a total of 452 articles from the six behavior analytic journals. We merged 46 duplicate articles and excluded 227 articles that fell outside of the publication date range. No studies were removed after limiting to peer review or English-only studies. One-hundred and seventy-nine studies remained for title and abstract and full-article screening. During this screening phase, 81 articles were excluded for not meeting inclusion criteria (e.g., not being an experimental study, not teaching FCRs or mands). Thus, a total of 98 articles were used to extract key FCR and mand training variables. Note: All percentages reported in the results reflect the number of *applications* (i.e., individual FCT or mand training instances), not the number of *studies*.

Across the 98 coded articles, 63 articles described FCT procedures, with a total of 250 individual applications of FCT included in the analysis. Thirty-five studies described mand training procedures, and a total of 104 applications of mand training were included in the analysis. We examined teaching procedures based on individual applications of either FCT or mand training so that we could accurately display participant-specific teaching modifications, if needed. Twenty-nine articles included individualized procedural modifications for at least one participant.

For non-mutually exclusive variables, percentages throughout the Results reflect the proportion of applications in which a given feature was present and may sum to greater than 100%.

Note: As no formal quality appraisal was conducted, this review does not present findings related to methodological rigor or risk of bias.

### 3.2. Participants Setting

Table 2 displays participant and setting information for both FCT and mand training studies. In studies evaluating FCT, participants spanned a broad age range. Specifically, 11.2% were between birth and 3 years, 40.0% were aged 4 to 6 years, and 39.6% were 7 to 12 years old. Adolescents aged 13 to 18 years comprised 6.8% of participants, while 2.4% were adults between 19 and 64 years. No participants were reported in the 65 years or older age group.

In mand training studies, the largest proportion of participants were children aged 4 to 6 years (49.0%), followed by those aged 7 to 12 years (21.1%), and birth to 3 years (9.6%). Adolescents aged 13 to 18 years represented 8.7%, adults aged 19 to 64 years accounted for 9.6%, and 1.9% of participants were 65 years or older.

Among participants in FCT studies, 76.0% were diagnosed with autism spectrum disorder (ASD) while 22.4% were diagnosed with developmental disabilities, including conditions such as Down syndrome or global developmental delay. An additional 12.0% were reported to have attention-deficit/hyperactivity disorder (ADHD). No participants were identified as having dementia or other degenerative conditions. Participants did not have a diagnosis in 3.2% of applications of FCT, and 4.8% of studies did not specify diagnostic status. Diagnoses categorized as “other” were reported for 31.2% of participants.

In mand training studies, 78.8% of participants were identified with ASD. Developmental disabilities were reported for 5.8%, ADHD for 1.9%, and dementia or other degenerative disorders for 1.9%. A diagnosis was not reported for 3.8% of participants, and 3.8% of studies did not specify diagnostic status. Diagnoses classified as “other” accounted for 11.5% of participants.

The majority of FCT studies were conducted in outpatient clinic settings (75.9%), followed by home settings (17.7%) and school environments (3.6%). Inpatient clinical settings were used in 0.8% of studies, and no studies were conducted in vocational programs. The setting was not reported or was unknown in 2.0% of cases.

Mand training studies showed greater variability in settings. Outpatient clinics were used in 39.8% of studies, home settings in 34.5%, and schools in 18.6%. No mand training studies were conducted in inpatient clinics or vocational programs. The setting was unspecified in 5.3% of studies.

Language assessment procedures were reported in 64.8% of FCT studies. Among these, 64.8% included descriptive language data (e.g., mean length of utterance), while standardized assessments were less common: 0.8% referenced individualized education programs (IEPs), 0.8% used the Peabody Picture Vocabulary Test (PPVT; Dunn, 2019), 1.6% used the Verbal Behavior Milestones Assessment and Placement Program (VB-MAPP; Sundberg, 2014), and 0.8% used the Early Echoic Skills Assessment (EESA; Esch, 2014). No studies employed the VB-MAPP Intraverbal Subtest (Sundberg, 2014), the Assessment of Basic Language and Learning Skills (ABLLS; Partington & Sundberg, 1998), the Assessment of Basic Language and Learning Skills—Revised (ABLLS-R; Partington, 2010), the Expressive One-Word Picture Vocabulary Test—Fourth Edition (EOWPVT-4; Martin & Brownell, 2011), the Expressive Vocabulary Test—Third Edition (EVT-3; Williams, 2019), or the Preschool Language Scale—Fifth Edition (PLS-5; Zimmerman et al., 2011). Language assessment procedures were not reported in 32.8% of applications.

In mand training applications, language assessments were reported in 94.2% of cases. Of these, 45.2% included descriptive information, 24.0% used the VB-MAPP, and 5.8% employed the EESA. Additional assessments included ABLLS or ABLLS-R (6.7%) and the EVT (2.9%). The PPVT, VB-MAPP Intraverbal Subtest, EOWPVT, and PLS were not used in any mand training applications. Language assessments were not reported in 16.3% of applications.

These data highlight notable differences in where and with whom FCT and mand training are implemented. While both target similar populations (primarily children with ASD), mand training appears more diverse in setting and assessment tools, potentially reflecting broader skill acquisition goals. These contextual distinctions are important when considering instructional delivery, which is addressed further in the discussion.

### 3.3. Behavior Reduction Information

Table 3 displays functional behavior assessment (FBA) type, response topographies, and functions of behavior targeted in FCT. FBAs were frequently used in FCT applications. 53.2% of applications reported conducting indirect assessments, 18.8% used direct assessments, and 97.2% included an experimental functional analysis component.

The most commonly targeted behavior topographies included aggression (76.8%), property destruction or disruption (54.8%), and self-injurious behavior (36.8%). Additional topographies targeted included negative vocalizations (22.8%), tantrums (11.6%), elopement (3.6%), and inappropriate mealtime behavior (1.2%). Surrogate behaviors were used in 5.2% of translational applications. No applications targeted stereotypy (0.0%).

In terms of behavior function, FCT interventions most frequently addressed tangible reinforcement (38.8%) and escape-maintained behavior (34.4%). Other reported functions included synthesized functions (19.2%), mand compliance (8.8%), attention (10.0%), access to rituals (3.2%), and automatic reinforcement (0.8%). In 8.8% of applications, the function of behavior was not specified.

### 3.4. Prompting Methods

**Figure 2:**
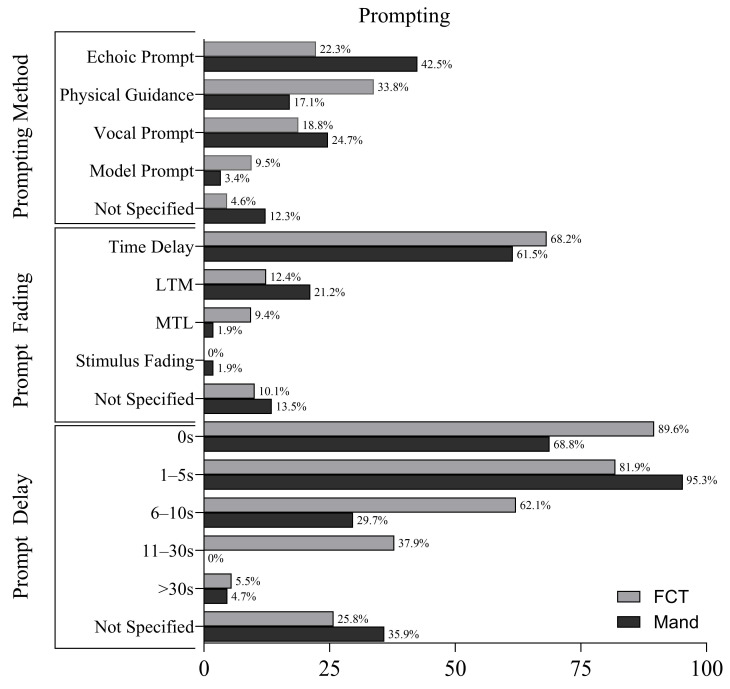
Prompting Method in FCT and Mand Training.

Figure 2 displays the prompting methods used in applications of FCT and mand training. Prompt delay procedures were widely used in both FCT and mand training applications. In FCT applications, 0 s prompt delays were reported in 89.6% of cases. Delays of 1–5 s were used in 81.9%, 6–10 s in 62.1%, 11–30 s in 37.9%, and delays greater than 30 s were used in 5.5% of applications.

Mand training applications similarly reported 0 s prompt delays in 68.8% of applications, followed by 1–5 s delays in 95.3%. Delays of 6–10 s were reported in 29.7% of cases, while no applications reported delays of 11–30 s. Delays longer than 30 s were used in 4.7% of applications.

In FCT applications, 68.2% of applications used time delay procedures. Least-to-most prompting was reported in 12.4%, most-to-least prompting in 9.4%, and the prompt fading procedure was not specified in 10.1% of applications.

In mand training, time delay procedures were used in 61.5% of applications. Least-to-most prompting was reported in 21.2%, most-to-least in 1.9%, and stimulus fading in 1.9%. The prompt fading procedure was not specified in 13.5% of applications.

With respect to specific prompt types, physical guidance was reported in 33.8% of FCT applications, echoic prompts in 22.3%, model prompts in 9.5%, and vocal prompts in 18.8%. Prompting methods were not reported in 4.6% of applications.

In mand training, echoic prompts were most common (42.5%), followed by vocal prompts (24.7%), physical guidance (17.1%), and model prompts (3.4%). Prompting procedures were not specified in 12.3% of applications.

Prompting strategies were largely similar across both interventions, though mand training more frequently included echoic prompts and longer delays. These similarities and differences may reflect each intervention’s underlying instructional priorities, particularly related to acquisition versus behavior suppression.

### 3.5. Establishing Operations

**Figure 3:**
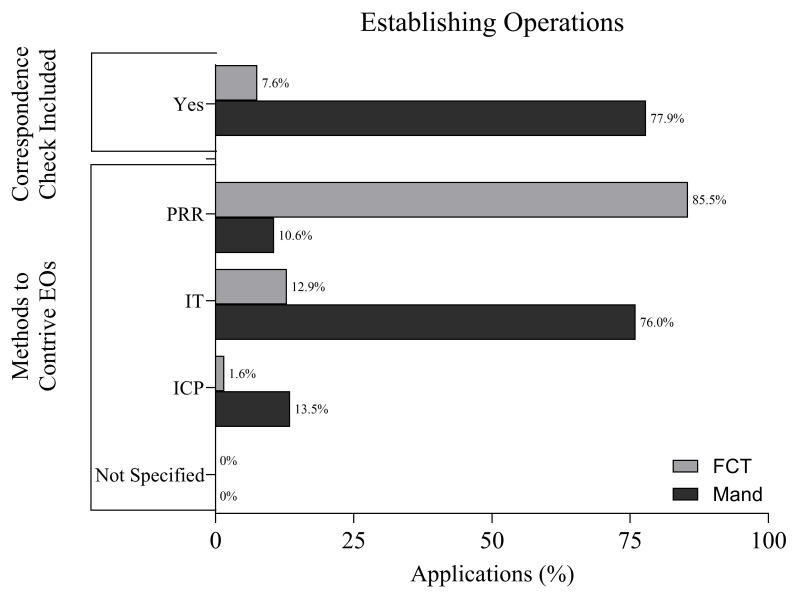
Establishing Operation (EO) Manipulations.

Figure 3 displays the methods for contriving EOs and if methods to check for correspondence between the EO and communication response were present. In FCT applications, the most common method for contriving EOs was programmed restriction of reinforcement, reported in 85.5% of applications. Incidental teaching was used in 12.9% of applications, and interrupted chain procedures were employed in 1.6%. No applications failed to report their EO manipulation procedures.

In mand training applications, incidental teaching was the predominant method, occurring in 76.0% of applications. This was followed by interrupted chain procedures (13.5%) and programmed restriction of reinforcement (10.6%). Again, no applications omitted reporting on EO manipulation.

In addition to methods to contrive EOs, communication training can include procedures to check for correspondence between the present EO and the response-specifying reinforcer. In applications of FCT, 7.6% included a method to check for correspondence and 92.4% applications did not check for correspondence. In mand training applications, however, 77.9% of applications incorporated methods to check for correspondence, while 22.1% of applications did not include these procedures.

Although both interventions involve manipulating establishing operations, FCT relied more on programmed restriction of reinforcers and did not check for correspondence, while mand training emphasized incidental teaching, naturalistic arrangements, and checking for correspondence between EOs and reinforcers. These patterns suggest differences in how motivational control is conceptualized and implemented, which is an issue explored in depth in the discussion.

### 3.6. Number of Responses Taught

**Figure 4:**
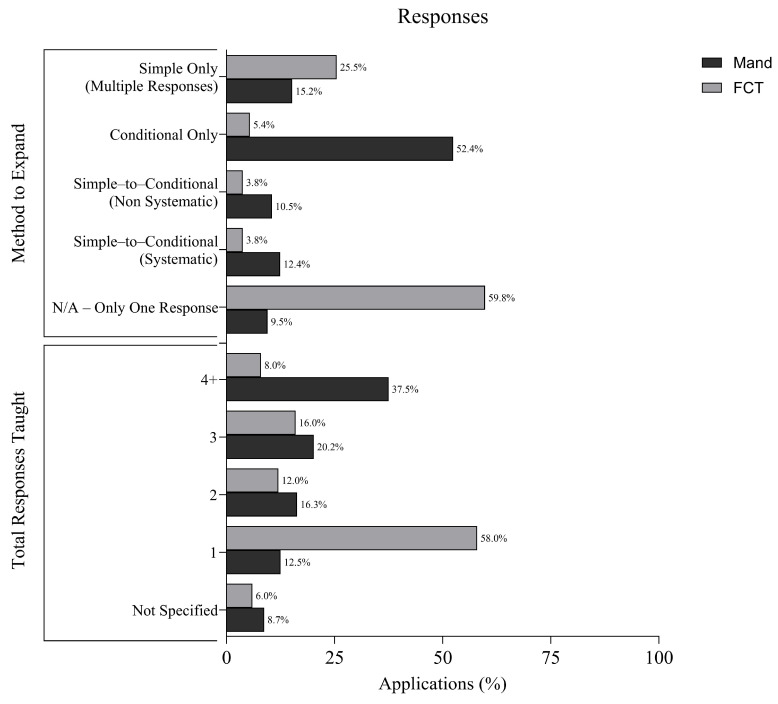
Response Information in FCT and Mand Training.

Figure 4 depicts information regarding number of responses taught and methods to increase the number of responses. Across FCT applications, the total number of communication responses taught varied. An FCR was taught in 58.0% of applications. Two FCRs were taught in 12.0% of applications, three FCRs in 16.0%, and four or more FCRs in 8.0%. The total number of responses targeted was not reported in 6.0% of applications.

In mand training applications, a broader range of responses was targeted. Four or more mands were taught in 37.5% of applications, three mands in 20.2%, two in 16.3%, and only one mand in 12.5% of cases. The number of responses taught was not specified in 8.7% of applications.

In FCT applications, 52.0% of applications did not attempt to expand the FCR repertoire beyond a single taught response. When repertoire expansion was implemented, 24.6% of applications taught each FCR in isolation as a simple discrimination. Additionally, 10.2% of applications began with one response and then used a systematic progression (e.g., simple-to-conditional discrimination), while 7.0% taught multiple responses individually first and then introduced conditional discrimination. Only 3.9% of applications targeted all responses from the outset. Methods for repertoire expansion were not reported in 2.3% of applications.

In mand training applications, 52.9% of applications targeted all mands from the beginning of instruction. Other approaches included teaching mands in isolation as simple discriminations (15.4%), or first teaching multiple simple responses and then introducing conditional discrimination (11.5%). An additional 10.6% began with one mand and used a systematic progression to expand the repertoire, such as systematically increasing the complexity of the discrimination (e.g., Akers et al., 2019). Only 9.6% of applications taught a single mand without attempting to expand the repertoire, and no applications failed to specify their method.

These data demonstrate a clear pattern in the two types of communication teaching. FCT applications often targeted a single communicative response, whereas mand training more frequently involved building broader repertoires and conditional discriminations. This distinction speaks to each intervention’s functional goals and has important implications for generalization and maintenance.

### 3.7. Session Arrangement and Mastery Criteria

**Figure 5:**
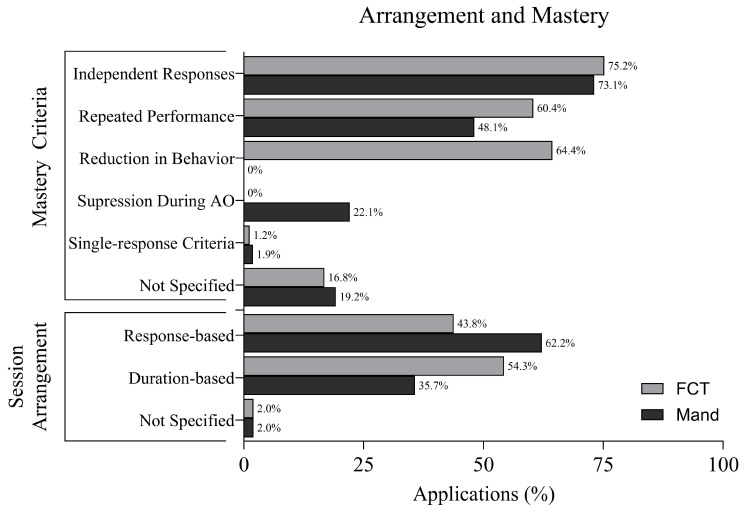
Session Arrangement and Mastery Criteria.

Figure 5 displays the session arrangements and mastery criteria in FCT and mand training applications. Among FCT applications, 54.3% used response-based session arrangements, while 43.8% used duration-based formats. The arrangement was not specified in 2.0% of applications.

In mand training, 62.2% of sessions were response-based, 35.7% were duration-based, and 2.0% did not specify the arrangement.

In FCT applications, 75.2% reported increases in independent responding as part of the mastery criteria, while 64.4% required reductions in challenging behavior. 60.4% required repeated performance, and 1.2% used a single-response criterion. No FCT applications included suppression of responding during AO trials, and 16.8% did not specify mastery criteria.

For mand training applications, 73.1% reported increases in independence, 48.1% required repeated performance, and 22.1% included suppression during AO trials. Single-response criteria were used in 1.9%, and 19.2% of applications did not specify mastery standards.

### 3.8. Response Topography and Selection Method

**Figure 6:**
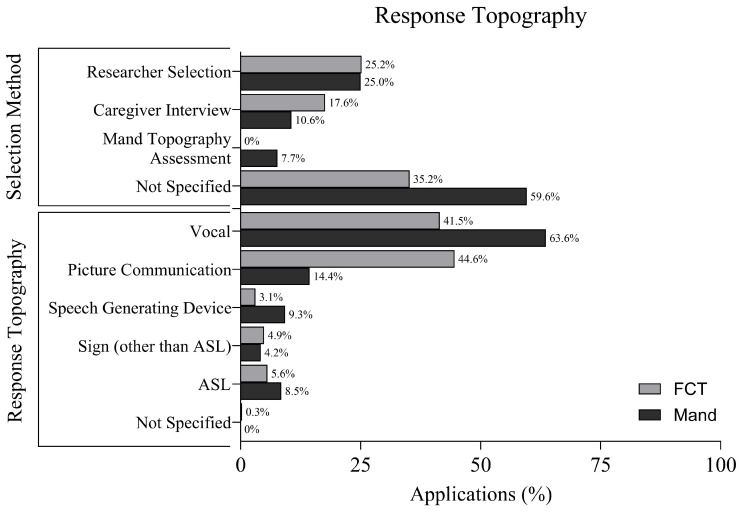
Response Topographies and Selection Method.

Figure 6 displays response topographies and methods for selecting response topographies. In FCT applications, the most frequently used response topographies were picture communication systems (44.6%) and vocal responses (41.5%). Other topographies included American Sign Language (ASL; 5.6%), sign language other than ASL (4.9%), and speech-output devices (3.1%). Only 0.3% of applications did not report the response topography.

In mand training applications, vocal responses dominated (63.6%), followed by ASL (8.5%), speech-output devices (9.3%), picture communication systems (14.4%), and other sign languages (4.2%). All applications specified the response mode.

In FCT applications, the method for selecting the FCR topography was not reported in 65.2% of applications. Therapist or researcher selection was documented in 26.0%, and caregiver interviews were used in 17.6% of cases. No applications utilized a mand topography assessment.

In contrast, mand training applications reported a mand topography assessment in 7.7% of cases. Therapist or researcher selection was used in 25.0%, caregiver interviews in 10.6%, and the method was not specified in 59.6% of applications.

### 3.9. Parameters of Reinforcement

**Figure 7:**
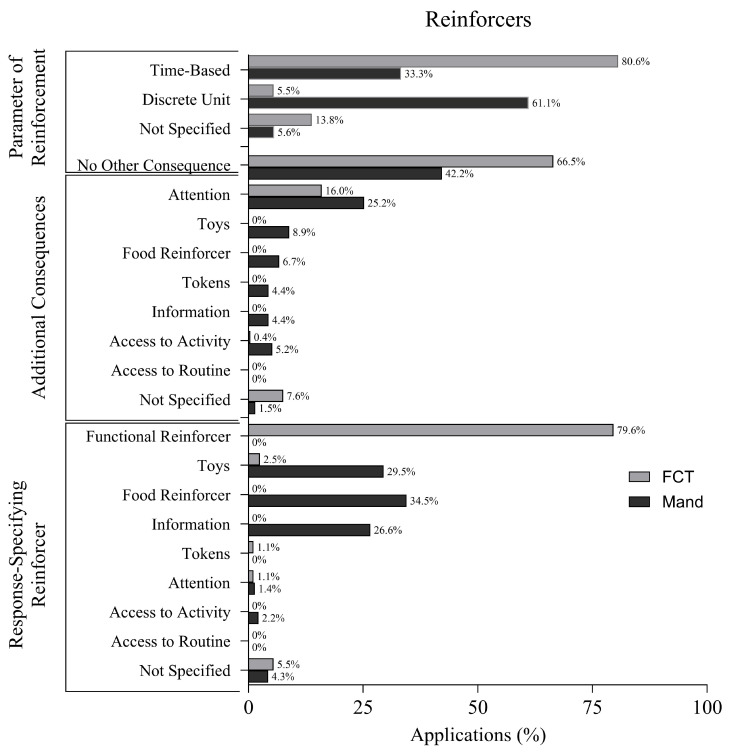
Types and Parameters of Reinforcers.

Figure 7 displays reinforcement information for FCT and mand training. In FCT applications, function-based reinforcers (as identified via functional behavior assessment) were delivered in 79.6% of applications. Non-function-based reinforcers were used in 10.2%, while toys were delivered in 2.5% of cases. Use of attention and tokens was reported in 1.1% each. No applications reported using snack reinforcers, information, access to routine, or access to activity. Reinforcer type was not specified in 5.5% of applications.

In mand training applications, a broader variety of reinforcers was observed. Snack reinforcers were used in 34.5%, toys in 29.5%, and information in 26.6% of applications. Attention was delivered in 1.4%, and access to activities in 2.2%. Function-based reinforcers were used in only 1.4% of applications. No applications reported using tokens, access to routine, or non-function-based reinforcers, and 4.3% of applications did not specify the reinforcer type.

In FCT applications, 66.5% did not implement any additional consequences beyond the primary reinforcer. Attention was delivered as an added consequence in 16.0% of applications, while toys and snack reinforcers were not used. Tokens and information were also absent. The consequence was not specified in 7.6% of cases. No applications reported using access to routines or access to activity.

In mand training, 42.2% of applications did not deliver additional consequences. However, attention was provided in 25.2%, toys in 8.9%, and snack reinforcers in 6.7%. Information and access to activity were reported in 4.4% and 5.2%, respectively. No applications included tokens or access to routines. Additional consequences were not specified in 1.5% of applications.

In FCT applications, 80.6% used a time-based reinforcement schedule, while 5.5% employed discrete unit reinforcement, such as a snack item or other consumable reinforcers. No applications reported using conditioned reinforcers. Reinforcement parameters were not specified in 13.8% of applications.

For mand training, 61.1% of applications used discrete unit reinforcement schedules, compared to 33.3% using time-based schedules. Parameters of reinforcement were not specified in 5.6% of applications.

Taken together, the findings illustrate both shared procedures and meaningful divergences in how FCT and mand training are implemented. These differences appear to align with each intervention’s primary goals of behavior reduction in FCT and skill acquisition in mand training. The following section explores these patterns and their implications for research and clinical practice.

## 4. Discussion

In this evaluation we examined factors related to teaching the FCR and mand in published literature over the past 10 years. Our goals were threefold: (1) to review teaching strategies for developing mand and FCR repertoires; (2) to analyze unique and shared teaching features; (3) to identify considerations for future research.

### 4.1. Teaching Procedures

#### 4.1.1. FCT

In the FCT literature, trial-based instructional arrangements were also the most commonly reported teaching format. Several studies referred to this as “FCT pre-training,” wherein the FCR was taught to mastery within discrete trials prior to implementation of the full FCT intervention. In these arrangements, the EO was presented, the response was evoked by the EO or prompted to occur in the presence of the EO, and the reinforcer was delivered contingent on the FCR. This format was typically used to ensure acquisition of the FCR before introducing the response into more naturalistic or extended sessions.

Trial-based instruction in FCT offers several practical and conceptual advantages. The controlled presentation of the EO and the clear response–reinforcer contingency promotes acquisition, particularly for learners with a limited verbal repertoire or a history of challenging behavior maintained by specific reinforcers. Additionally, these sessions allow clinicians to verify that the FCR is emitted under appropriate motivational conditions prior to embedding it into more complex or variable contexts.

Duration-based arrangements may better approximate natural contexts than trial-based formats, but they also introduce challenges related to motivational control and relevant EOs. For example, during extended sessions, the EO may fluctuate across time, and prompted responses may occur under varying motivational conditions—potentially weakening the functional relation between the EO and the FCR (see Fisher et al., 2018, for related challenges with maintaining motivational control in FCR interventions).

From a conceptual standpoint, the timing of reinforcement delivery relative to the EO is critical. If prompting or reinforcement occurs when the EO is absent, or only partially in effect, the FCR may come under stimulus control instead of motivational control. That is, the learner may emit the response in the presence of irrelevant stimuli, rather than under the control of the EO. Reinforcing a response in the absence of motivation may weaken the contingency between the EO and the FCR, reducing the functional relevance of the communicative behavior (Michael, 1993). This is especially pertinent in FCT, where the EO is deliberately manipulated to prevent the occurrence of challenging behavior.

Prior to initiating FCT, researchers typically conducted a functional analysis (FA) or similar experimental assessment to identify the reinforcer(s) maintaining the individual’s challenging behavior. The most common methods used in the studies reviewed included multielement functional analyses (Iwata et al., 1994) and variations such as the interview-informed synthesized contingency analysis (IISCA; Hanley et al., 2014). The results of these assessments served as the foundation for selecting the functional reinforcer(s) to incorporate into the FCT procedures.

Function-based treatment is central to the efficacy of FCT, and identification of the relevant functional reinforcer is critical to the development of a communication response that is both effective and socially meaningful. In the reviewed studies, reinforcers included access to attention, tangible items, escape from demands, or combinations of these variables. The selected FCR was then taught to replace the challenging behavior and contact the identified reinforcer under similar EOs.

By conducting an FA prior to treatment, researchers were able to establish a clear functional relationship between the EO and the reinforcer maintaining the behavior. This relationship creates the opportunity for the direct replacement of the challenging behavior with a functionally equivalent communication response. In this way, FCT relies on experimentally derived evidence to inform treatment design and ensure that the communication response contacts the same reinforcement that previously maintained challenging behavior.

In the reviewed literature, contriving the EO often involved temporarily withholding or interrupting access to the identified reinforcer. This procedure aligns with strategies described in mand training, such as incidental teaching arrangements in which access to the reinforcer is blocked to evoke the relevant response (Frampton et al., 2024b). However, the application of EO contrivance in FCT often differed from that in mand training.

In many FCT studies, researchers frequently employed a 0 s prompt delay and prompted the FCR immediately following the presentation of the EO. This approach was particularly common in early phases of instruction or during pre-training sessions, and was likely included to reduce the likelihood of challenging behavior by limiting the time in the EO. Researchers have demonstrated that shorter EO durations can result in more rapid reductions in challenging behavior and fewer behavior bursts (DeRosa et al., 2015; Fisher et al., 2018). These findings support the use of immediate prompting in situations where the EO has been reliably identified via functional analysis and the risk of challenging behavior is high.

The underlying assumption in FCT is that the EO is present when the reinforcer is removed or withheld, based on the functional relationship previously demonstrated during assessment. In this context, prompting the FCR immediately after EO presentation may allow for acquisition of the response without extended exposure to the EO, thereby minimizing the probability of challenging behavior.

Recent research has also explored alternative strategies for verifying EO presence in FCT. One possible method for identifying the presence of an EO is by looking at the presence of challenging behavior. The presence of challenging behavior likely indicates the presence of an EO; therefore, prompting an FCR contingent on challenging behavior could help FCRs come under the appropriate motivational control. Landa et al. (2022) examined whether prompting an FCR following the occurrence of challenging behavior would affect treatment outcomes. Their findings suggested that prompting communication after challenging behavior did not produce maladaptive behavior chains and, in many cases, supported successful acquisition of the FCR. These findings highlight the potential value of moment-based EO identification, particularly in cases where traditional EO manipulation strategies may not be sufficient or precise.

#### 4.1.2. Mand Training

Across all included applications, trial-based instruction was the most commonly used arrangement. Mand training lends itself to trial-based instruction because the trial-based arrangements allow for a clear presentation of the EO, the opportunity for the response, and a discriminable reinforcer. This format provides learning opportunities in which the EO, behavior, and consequence can be isolated and systematically arranged. In doing so, trial-based instruction creates conditions under which clinicians can evaluate whether the mand occurs in the presence of the relevant EO and is maintained by the delivery of the response-specifying reinforcer.

Although trial-based formats offer tight experimental control, approximately one-third of the mand training applications included in our analysis used duration-based sessions. These sessions were more commonly observed when the goal of instruction was to modify pre-existing mand repertoires (e.g., increase words per mand; Shillingsburg et al., 2020) or train implementers on the instructional procedures (e.g., Lerman et al., 2015; Tsami et al., 2019). Duration-based sessions often included periods of natural or sustained access to the EO, during which opportunities for manding and reinforcement were embedded throughout the session.

While duration-based arrangements allow for less precise control over individual teaching trials, they may offer greater ecological validity. Unlike trial-based formats, which isolate learning opportunities, duration-based sessions more closely reflect the dynamic and variable presentation of motivational conditions in natural environments. These features may facilitate generalization by allowing the learner to emit responses under more naturalistic stimulus conditions and across varying EO durations.

Several methods were used across studies to contrive EOs during mand training. In a recent conceptual analysis, Frampton et al. (2024b) described three primary strategies for contriving EOs in clinical practice: incidental teaching arrangements, interrupted chain procedures, and programmed deprivation. These procedures were commonly observed in our review and provide a useful framework for categorizing EO manipulation in mand training.

Incidental teaching arrangements were the most commonly used method. These procedures involved presenting the reinforcer in the environment while temporarily withholding access. In some instances, the reinforcer was placed in view but out of reach of the learner, while in others it included reinforcer interruption by allowing brief access to the reinforcer before removing the reinforcer. In both cases, the EO was manipulated by disrupting access to an available reinforcer, thereby increasing the probability of a mand. These strategies may be especially effective when the goal is to evoke spontaneous manding in naturalistic contexts, and they are widely used across both research and applied settings.

Interrupted chain procedures were also reported in a subset of applications. In these arrangements, the teaching environment was structured such that a pre-established behavior chain is disrupted, typically by removing or altering one of the critical components required to complete the chain. For example, during an activity like making a craft, a necessary material might be missing or inoperable, thereby creating an EO for the learner to request the missing item. Conceptually, this approach functions by establishing a transitive conditioned motivating operation (CMO–T), in which the value of an otherwise neutral stimulus increases because it is needed to access the terminal reinforcer (Michael, 1993). Although this procedure was less frequently reported, it represents a powerful method for contriving complex motivational conditions and for establishing mands that are contextually embedded within meaningful routines.

Programmed deprivation was the third EO manipulation strategy observed. In this method, access to a reinforcer is systematically restricted prior to the instructional session to increase the value of that reinforcer when it becomes available. This may include closed economy arrangements, in which the reinforcer is only available during specific teaching sessions, or natural periods of deprivation, such as targeting food-related mands prior to lunch. While this strategy relies on naturally occurring fluctuations in motivation, it may also be used proactively to strengthen the EO and improve the likelihood of appropriate manding during instruction.

Together, these procedures represent conceptually distinct but functionally similar methods for establishing motivating conditions under which mands are taught. Each strategy reflects a different balance of environmental arrangement, timing, and learner experience, and their selection in clinical practice may depend on the instructional context, the complexity of the repertoire, and the developmental level of the learner.

Taken together, the literature suggests that FCT relies more heavily on systematic EO contrivance and reinforcement contingencies derived from experimental functional analyses. However, unlike in mand training, FCT procedures minimize time in the EO, presumably to decrease the risk associated with increased challenging behavior with increased EO durations (see below for further discussion). This distinction reflects the competing priorities of the procedures; whereas mand training often focuses on skill acquisition and generalization, FCT balances skill development with immediate behavior reduction in high-risk situations.

### 4.2. Comparison of Procedures

#### 4.2.1. Similarities

FCT and mand training share a number of core instructional strategies and conceptual features. One of the most consistent similarities observed across the reviewed literature was the use of short prompt delays as a primary teaching strategy. Both procedures commonly implemented a brief prompt delay at the onset of implementation, followed by systematic increases in the delay as the learner demonstrated independent responding. Prompting strategies typically included physical guidance and echoic prompts, and both procedures emphasized errorless learning models in which prompts were delivered proactively rather than reactively.

Another commonality between FCT and mand training was the use of incidental teaching procedures, particularly those involving the withholding or interruption of access to the reinforcer. This strategy was used to contrive the EO and increase the likelihood that the target response would be evoked under the appropriate motivational conditions. Although there were difference in the frequency and complexity of EO manipulations, both relied on the principle of temporarily limiting access to an identified reinforcer to create periods of deprivation.

Finally, both procedures typically included delivery of the response-specifying reinforcer and minimal inclusion of additional, arbitrary consequences. In most applications, the reinforcer delivered was directly related to the content of the FCR or mand (e.g., access to a tangible item, escape from demand, attention), with generalized reinforcement such as praise infrequently reported as a programmed component of instruction. This approach preserves the discriminative and motivational control of the response and helps prevent the dilution of the reinforcement contingency. From a conceptual standpoint, the delivery of multiple consequences for a single response (e.g., praise plus tangible) could interfere with the specificity of the response–reinforcer relation and introduce ambiguity in motivational control.

#### 4.2.2. Differences

Despite these shared features, several key differences emerged between FCT and mand training, particularly in terms of duration of exposure to the EO. These procedural differences appear to reflect underlying differences in treatment goals and risk profiles associated with each procedure.

In mand training, the most common prompt delay was in the range of 1–5 s. This delay allowed the learner to remain in the EO briefly before prompting, which may support stronger motivational control over the response. The longer prompt delay creates an opportunity for the learner to emit the response independently under the control of the relevant EO, while still allowing for instructional support (e.g., controlling prompt) if the response does not occur. Because mand training is often used in contexts where challenging behavior is not a primary concern, the procedures can afford to allow the EO to build gradually without an immediate prompt.

In contrast, FCT applications more commonly used a 0 s prompt delay, particularly during the onset of treatment. This approach reflects the priority of rapid behavior reduction in FCT. Given that FCT is often implemented in response to high-intensity or dangerous challenging behavior, extended exposure to the EO may result in increased risk of behavioral escalation. Prompting the FCR immediately after EO presentation minimizes time in the EO and reduces the likelihood that the challenging behavior will occur before the replacement response is emitted. This strategy is consistent with prior findings that shorter EO exposure during early FCT can reduce bursts of behavior and promote faster acquisition of the communication response (DeRosa et al., 2015; Fisher et al., 2018).

These differences underscore the differing priorities that define each procedure. FCT prioritizes immediate functional replacement of challenging behavior to ensure safety and reduce disruption. In doing so, FCT often limits the learner’s time in the EO and relies on prompt-heavy instruction initially. Mand training, on the other hand, prioritizes skill acquisition and repertoire expansion. The teaching procedures in mand training are more likely to allow for extended time in the EO and may incorporate more nuanced EO manipulations or generalization strategies. As a result, the two procedures reflect distinct applications of the same behavioral principles, tailored to different treatment goals.

In addition to differences in EO exposure and prompting strategies, FCT and mand training procedures also diverged in terms of the number of responses targeted and the use of conditional discrimination. Mand training interventions were more likely to begin with multiple communication responses and to incorporate procedures that taught learners to discriminate between EOs to emit functionally appropriate mands. In fact, mand training studies in the review commonly initiated instruction with conditional discrimination training rather than first establishing isolated mands and later adding complexity (e.g., Lerman et al., 2015; Sellers et al., 2016). These studies frequently arranged teaching trials that presented varied EOs or contrived scenarios requiring the learner to select the correct mand based on their motivational state. This early emphasis on conditional discrimination reflects an instructional focus on developing flexible, functionally appropriate communication repertoires under motivational control.

In contrast, FCT interventions more commonly focused on teaching a single FCR corresponding to the reinforcer identified through a functional analysis (e.g., DeRosa et al., 2015; Fisher et al., 2018). These studies prioritized the rapid acquisition of an alternative response to replace challenging behavior, typically emphasizing the functional relation between the FCR and the maintaining consequence over broader repertoire development. When additional FCRs were introduced, they were often taught sequentially rather than concurrently, and each was associated with a specific antecedent arrangement or discriminative stimulus (e.g., colored cards, therapist instructions), rather than the learner’s motivational state. While these strategies were effective for reducing challenging behavior and promoting clear stimulus control, they did not commonly include procedures to teach conditional discriminations based on motivational control. That is, learners were rarely required to assess which EO was present and then emit the appropriate FCR from among multiple possible responses. Conditional manding arrangements that require discrimination across multiple motivational contexts have been demonstrated in the literature (e.g., Akers et al., 2019), but were uncommon among studies reviewed here. This distinction may reflect the differing priorities of FCT (e.g., safety and behavior reduction) where rapid acquisition, low error rates, and response efficiency are often favored over complexity and flexibility in early instruction (e.g., Tiger et al., 2008; Greer et al., 2016).

### 4.3. Considerations for Future Research

#### 4.3.1. Contriving EOs

Although both FCT and mand training rely heavily on EO manipulation, our review suggests a need for greater conceptual and procedural clarity in how EOs are contrived and verified, particularly in FCT. While incidental teaching strategies, such as temporarily withholding or interrupting access to reinforcers, were common across both procedures, many studies did not report methods for confirming that the EO was actually present at the time of response prompting. This raises questions about the consistency of motivational control and durability of the response.

Future research should examine strategies that ensure EOs are present at the time of prompting, rather than assuming EO presence based solely on reinforcer removal. For instance, studies could evaluate the effectiveness of incorporating indicating responses, such as reaching, pointing, or looking at a reinforcer, prior to prompting an FCR. These responses may serve as observable indicators of motivation and could be used to confirm the presence of an EO before instruction occurs. Relatedly, few FCT applications explicitly assess responding under abolishing operation (AO) conditions, which limits conclusions about whether FCRs are under discriminated motivational control. Incorporating procedures that evaluate responding across both EO and AO conditions may strengthen interpretations of FCR repertoires. This approach has been emphasized in recent clinical tutorials on mand training (Frampton et al., 2024a) but has received limited attention in FCT applications.

Additionally, future work should explore the potential for interrupted chain procedures to be adapted for FCT contexts. While this strategy is more commonly used in mand training, it reflects broader class of instructional arrangements (e.g., multiple schedules) that systematically contrive complex motivational conditions. Interrupted chain procedures offer a conceptually rich method for contriving CMO–Ts (Michael, 1993) by removing or disabling components necessary to complete a preferred routine. For example, if a learner typically requests a tablet, the EO could be contrived by presenting a tablet that is out of battery, locked, or missing an accessory. These modifications increase the value of the missing item or information and create an opportunity for the learner to emit an FCR under a more complex motivational condition and improving generalization. Although these procedures have been used in mand training to establish generative responding, their utility in FCT remains underexplored.

Finally, future research may benefit from examining qualitative features of reinforcement as they relate to EO strength. For example, a brief or low-quality break from instruction may increase the EO for a longer or more engaging break. Understanding how qualitative variation in reinforcer delivery affects EO strength and response allocation could improve the precision of EO manipulation in both FCT and mand training (Brown et al., 2021).

#### 4.3.2. Correspondence Between the EO and the Response

In addition to verifying EO presence, future research should continue to investigate the role of correspondence between the EO and the communication response. In mand training, it was common for studies to include procedures that ensured correspondence through either pre-response indicators (e.g., indicating responses) or post-response checks (e.g., selection of the requested item when offered a choice between it and an alternative). These strategies help confirm that the response was emitted under the control of the EO, rather than under unrelated environmental cues or generalized reinforcer availability.

This level of correspondence is critical for building a communication repertoire that is functionally meaningful and under appropriate motivational control (Skinner, 1957). Without such procedures, there is a risk that the communication response is maintained by reinforcement, but not under the control of motivational conditions for which it was intended. For example, if a learner is taught to request attention and tangible items are simultaneously removed, the learner may emit the attention FCR even when the EO is for the tangible item. Attention may reduce the overall level of challenging behavior due to its substitutability, but it does not satisfy the EO for the preferred item. This mismatch can weaken motivational control and limit generalization across settings or stimulus conditions.

McGill (1999) discussed this issue in terms of reinforcer substitutability, suggesting that while some reinforcers may reduce problem behavior in the short term, they do not establish the necessary control over the functional reinforcer. Over time, this lack of correspondence can result in confusion, inconsistent responding, or reemergence of challenging behavior when the reinforcer no longer functions as a substitute.

Future research should prioritize procedures that increase the likelihood of correspondence between the EO and the communication response. This includes identifying observable indicators of motivation, using consequence-based correspondence checks, designing instructional procedures that maintain specificity of the response–reinforcer relation, and incorporating AO trials into research preparations. Such work may strengthen motivational control, promote generalization, and reduce the likelihood of relapse or treatment failure.

Notably, correspondence checks were rarely included in the FCT literature reviewed in the current study. One possible reason for this omission is the practical challenge of implementing such procedures without inadvertently increasing the value of the EO (e.g., introducing a delay between the FCR and reinforcement) or creating a new EO (e.g., presenting additional demands that increase the value of escape). As a result, clinicians may de-prioritize correspondence checks to preserve treatment integrity, minimize response effort, and maintain the effectiveness of reinforcement contingencies.

Behavior analysis already has well-established behavioral technologies for measuring consumption, avoidance, and engagement, particularly within preference assessments and competing stimulus assessments. Measures such as duration of contact, latency to approach, or active rejection have been used to evaluate the reinforcing value of stimuli and the extent to which they compete with problem behavior (e.g., Piazza et al., 1996). Although these methods have typically been used to identify effective reinforcers, they could also be repurposed during reinforcement intervals in FCT as indirect indicators of EO satisfaction. For example, sustained consumption or high levels of engagement with a reinforcer following an FCR may suggest that the reinforcer matched the EO, whereas low engagement, avoidance, or continued challenging behavior may signal that the EO remains intact. While this application has not been systematically evaluated, the necessary tools already exist. Future research should explore how responding during reinforcement intervals can serve as practical correspondence checks; checks to detect residual EOs, refine teaching procedures, and prevent potential weakening of motivational control.

#### 4.3.3. Number of Responses Taught

Another critical area for future research involves the number of responses targeted during instruction. Across both FCT and mand training, many studies taught only a single FCR or mand during intervention. While this approach may be efficient for achieving initial treatment goals—particularly behavior reduction in the case of FCT—it raises important questions about the long-term durability and flexibility of the acquired repertoire.

Teaching a single FCR may be sufficient in highly controlled contexts, but it may not generalize well across situations where different reinforcers are available, the EO shifts, or stimulus conditions vary. In such cases, the limited response set may restrict the learner’s ability to access reinforcement through appropriate communication and increase the likelihood of response failure. These challenges are particularly salient in FCT, where the initial FCR can function as an omnibus request to access multiple reinforcers (e.g., “my way”). While this strategy can lead to rapid suppression of challenging behavior, it may fail to establish motivational control for isolated contingencies.

Future research should examine strategies for teaching multiple FCRs from the outset or incorporating response differentiation over time. Studies might compare outcomes when starting with a simple-to-conditional progression versus beginning instruction within a conditional discrimination format, where the learner must select from multiple responses depending on the specific EO. Prior research in skill acquisition suggests that learners can benefit from early exposure to more complex discriminations, which may lead to more durable and generalized responses (Grow & LeBlanc, 2013). Recent research on mand topography assessments (e.g., Kunnavatana et al., 2018; Livingston et al., 2024; Ringdahl et al., 2009) may provide a promising direction for the use of multiple FCRs in the treatment of challenging behavior. In the mand topography assessment, clinicians teach multiple topographies of the FCR (e.g., card exchange, speech-generating device) and systematically evaluate the learner’s preference for FCR modalities. By teaching multiple FCRs, the authors may promote greater generalization of communicative responses and increased durability of the FCR repertoire. Specifically, exposure to extinction across multiple FCR forms may mitigate relapse when reinforcement for a particular response is unavailable, while access to communicative options allows learners to be flexible in responding if one form does not contact reinforcement.

Incorporating multiple FCRs at the outset of teaching also provides opportunities to strengthen the relation between specific EOs and specific responses, thereby improving motivational control and increasing the precision of communication. In clinical settings, one practical strategy may involve introducing additional FCRs once the initial response is acquired and stable, using procedures such as differential reinforcement of alternative behavior in combination with noncontingent reinforcement. This approach may help maintain lower levels of challenging behavior while promoting a broader and more flexible communication repertoire.

In summary, although teaching a single FCR can be effective in the short term—particularly when the priority is behavior reduction—future work should explore methods for expanding response sets and promoting response selection that aligns more closely with dynamic motivational conditions. Such approaches may plausibly support generalization and the development of a more generative and functional communication repertoire, though this relation shas not been systematically evaluated in the existing mand training literature.

### 4.4. Limitations

While this review identified several key differences and shared features between FCT and mand training procedures, several limitations should be acknowledged when interpreting the findings.

First, the scope of the literature search was restricted to articles published between 2014 and 2024 and limited to six peer-reviewed journals with a primary focus on behavior analytic research. Although this strategy ensured a manageable and current dataset, it also limited the historical breadth of the review. Foundational or seminal work published prior to 2014 may contain detailed descriptions of teaching procedures, EO manipulation strategies, or mand development trajectories that were not captured in this review. It is also possible that more recent publications assume certain procedural steps (e.g., EO contrivance) are common knowledge and therefore not described in sufficient detail. As a result, some of the procedural gaps identified in this review may reflect limitations in reporting rather than true absence of these components in clinical application.

Second, the coding categories used to evaluate EO manipulation did not fully align with emerging conceptual frameworks in the literature. For example, Frampton et al. (2024b) proposed a set of EO contrivance strategies (i.e., incidental teaching, interrupted chains, and programmed deprivation) that offer a conceptually grounded method for organizing instructional procedures. The authors indicate that multiple EO contrivance strategies may be incorporated at once; however, our focus was on the primary contrivance method. As a result, our analysis may have obscured subtle differences in instructional design or EO control that would have been more clearly identifiable with a conceptually aligned coding system. Future work should consider adopting and validating such frameworks in systematic reviews and meta-analyses to improve the precision of procedural comparisons.

Finally, the perspective of the research team may have shaped the review process in meaningful ways. Our team’s primary area of expertise lies in behavior reduction and the treatment of challenging behavior, and as such, many of the search terms, coding decisions, and interpretations were informed by a functional analysis framework. Although every effort was made to approach the literature objectively, it is possible that important features of mand training, particularly those related to early language development, generative verbal behavior, or instructional design, were underrepresented due to disciplinary blind spots. Future reviews may benefit from interdisciplinary collaboration among researchers with expertise in verbal behavior, speech-language pathology, and developmental psychology.

Despite these limitations, this review highlights critical procedural and conceptual differences in how communication responses are taught across two widely used behavior analytic interventions. Addressing the identified gaps in future research may help improve instructional precision, enhance treatment outcomes, and advance our understanding of verbal behavior development in applied contexts.

This scoping review identified distinct instructional priorities across FCT and mand training literature. While FCT studies emphasized response efficiency and behavior reduction, mand training research more often targeted expansion of communication repertoires. These distinctions were reflected in prompting procedures, mastery criteria, and response topographies. These findings fulfill the review’s objectives by identifying the shared and unique features of FCT and mand instruction and highlighting areas for future research to bridge instructional strategies.

## Figures and Tables

**Table 1:** Boolean Search Strategy.

PsycInfo Search Strategy	ERIC Search Strategy
(“functional communication training” OR “functional communication response” OR “functional equivalence training” OR “mand” OR “mand training” OR “mand model” OR “teaching mands” OR “picture exchange communication system” OR pecs OR fct OR (funct* N/5 communic*) OR (pictur* N/5 communic*)) AND (“applied behavior analysis” OR “verbal behavior” OR “behavior analysis” OR “behavior analyst” OR “behavior modification” OR “behaviorism” OR “behaviorist” OR ((behav*) N/5 (therap* OR instruct* OR educ* OR profess* OR manpower)) OR (DE “Behavior Therapy” OR DE “Behavioral Health Services” OR DE “Therapists”)) AND (JN “Journal of Applied Behavior Analysis” OR JN “Behavior Analysis in Practice” OR JN “The Analysis of Verbal Behavior” OR JN “Behavior Modification” OR JN “Behavioral Interventions” OR JN “Behavior Analysis: Research and Practice”)	(“functional communication training” OR “functional communication response” OR “functional equivalence training” OR mand OR “mand training” OR “mand model” OR “teaching mands” OR “picture exchange communication system” OR pecs OR fct OR (“functional communication”) OR (“picture communication”)) AND (“applied behavior analysis” OR “verbal behavior” OR “behavior analysis” OR “behavior analyst” OR “behavior modification” OR “behaviorism” OR “behaviorist” OR (behavior* AND (therapy OR instruction OR education OR professional OR workforce))) AND (SO “Journal of Applied Behavior Analysis” OR SO “Behavior Analysis in Practice” OR SO “The Analysis of Verbal Behavior” OR SO “Behavior Modification” OR SO “Behavioral Interventions” OR SO “Behavior Analysis: Research and Practice”)

*Note.* An asterisk (*) indicates truncation to capture multiple word endings (e.g., *communicat* retrieves *communication*, *communicating*, *communicative*). The proximity operator *N/5* specifies that terms must appear within five words of each other.

**Table 2:** Participant and Setting Information.

	FCT	Mand
Age		
Birth-3 years	28 (11.2%)	10 (9.6%)
4–6 years	100 (40.0%)	51 (49.0%)
7–12 years	99 (39.6%)	22 (21.1%)
12–18 years	17 (6.8%)	9 (8.7%)
19–64 years	6 (2.4%)	10 (9.6%)
65 years +	0 (0%)	2 (1.9%)
Diagnosis		
Autism	190 (76.0%)	82 (78.8%)
Developmental disability	56 (22.4%)	6 (5.8%)
ADHD	30 (12.0%)	2 (1.9%)
Neurodegenerative disease	0 (0%)	2 (1.9%)
No diagnosis	8 (3.2%)	4 (3.8%)
Other	78 (31.2%)	12 (11.5%)
Not reported/unknown	12 (4.8%)	4 (3.8%)
Setting		
Clinic (outpatient)	189 (75.9%)	45 (39.8%)
Clinic (inpatient)	2 (0.8%)	0 (0%)
School	9 (3.6%)	39 (34.5%)
Home	44 (17.7%)	21 (18.6%)
Vocational program	0 (0%)	0 (0%)
Not reported/unknown	5 (2.0%)	6 (5.3%)
Language Assessment		
Descriptive Information	152 (60.8%)	47 (45.2%)
Information from IEP	2 (0.8%)	0 (0%)
PPVT ^a^	2 (0.8%)	6 (5.8%)
VB-MAPP ^b^	4 (1.6%)	25 (24.0%)
EESA ^c^	2 (0.8%)	6 (5.8%)
VB-MAPP (Intraverbal Subtest)	0 (0%)	7 (6.7%)
ABLLS ^d^ or ABLLS-R ^e^	0 (0%)	3 (2.9%)
EOWPVT ^f^	0 (0%)	0 (0%)
EVT ^g^	0 (0%)	0 (0%)
PLS ^h^	0 (0%)	0 (0%)
Not Reported	82 (32.8%)	17 (16.3%)

*Note.* The values in the FCT and Mand columns indicate the total frequency in the category and the percentage of the category, in parentheses. ^a^ PPVT = Peabody Picture Vocabulary Test. ^b^ VB-MAPP = Verbal Behavior Milestones Assessment Placement Program. ^c^ EESA = Early Echoics Skills Assessment. ^d^ ABLLS = Assessment of Basic Language and Learning Skills. ^e^ ABLLS-R = Assessment of Basic Language and Learning Skills—Revised. ^f^ EOWPVT = Expressive One Word Picture Vocabulary Test. ^g^ EVT = Expressive Vocabulary Test. ^h^ PLS = Preschool Language Scale.

**Table 3:** Behavior Reduction Information.

	Frequency
FBA Components	
Indirect Assessment	133 (53.2%)
Direct Assessment	47 (18.8%)
Experimental Analysis	243 (97.2%)
Topographies Targeted in Intervention	
Aggression	192 (76.8%)
Self-Injurious Behavior	92 (36.8%)
Disruptions/Property Destruction	137 (54.8%)
Negative Vocalizations	57 (22.8%)
Tantrums	29 (11.6%)
Inappropriate Mealtime Behavior	3 (1.2%)
Elopement	9 (3.6%)
Surrogate of Behavior (e.g., Translational Research)	13 (5.2%)
Stereotypic Behavior	0 (0%)
Function Targeted in Intervention	
Tangible	97 (38.8%)
Attention	25 (10.0%)
Escape	86 (34.4%)
Automatic	2 (0.8%)
Synthesized	87 (19.2%)
Social Control	22 (8.8%)
Access to Rituals	8 (3.2%)
Not Specified	22 (8.8%)

## Data Availability

All accessible data are contained in the manuscript.

## Abbreviations

The following abbreviations are used in this manuscript:

MO	Motivating Operation
EO	Establishing Operation
AO	Abolishing Operation
FCR	Functional Communication Response
FCT	Functional Communication Training
PRISMA	Preferred Reporting Items for Systematic Reviews and Meta-Analysis
IOA	Interobserver Agreement
ASD	Autism Spectrum Disorder
ADHD	Attention Deficit/Hyperactivity Disorder
IEP	Individualize Education Plan
PPVT	Peabody Picture Vocabulary Test
VB-MAPP	Verbal Behavior Milestones Assessment and Placement Program
EESA	Early Echoic Skills Assessment
ABLLS	Assessment of Basic Language and Learning Skills
ABLLS-R	Assessment of Basic Language and Learning Skills—Revised
EOWPVT	Expressive One-Word Picture Vocabulary Test
EVT	Expressive Vocabulary Test
PLS	Preschool Language Scale
FBA	Functional Behavior Assessment
ASL	American Sign Language
CMO-T	Transitive Conditioned Motivating Operation
FA	Functional Analysis
IISCA	Interview-Informed Synthesized Contingency Analysis

## Appendix A

### Coding Instructions

**Thank You for Your Help with Coding Articles for This Scoping Review!**

This review examines the teaching procedures used in functional communication training (FCT) and mand training. Each article requires the completion of two forms:

1. **FCT/Mand Training-Article Info form**
2. **FCT/Mand Training-Participant Info form**

**Step 1: Complete the Article Info Form**

The first section of this form determines whether the article meets the inclusion criteria.

- If the article does **not** meet the criteria (indicated by an answer containing “(exclude)”), answer “yes” to the final question, submit the response, and stop there.
- For excluded articles, **do not** complete the Participant Info form.

If the article is included, proceed with the remainder of the Article Info form:

- Provide general information about the article, such as the **author**, **publication date**, and **title**.

∘ **Tip:** Copy the article title after typing it into the form. You’ll need to paste it exactly as written into the Participant Info form for consistency.

- Answer questions about the **procedures**:

∘ If all participants had the same procedure for a given component, select that component (or components).
∘ If procedures differed among participants for a given component, select **“Procedure Varied By Participant.”**

**Step 2: Complete the Participant Info Form**

**For each participant in the study, you’ll need to submit one Participant Info form.**

- Paste the article title (copied from the Article Info form) into the form to ensure consistency. This helps organize the data.
- Provide information specific to each participant. For example, if the study had five participants, you will submit five separate Participant Info forms.

**When answering the question, “Did you select ‘Procedure Varied by Participant’ in the Article Info survey?”**

- Select “Yes” if you indicated the procedures varied by participant at any point when completing the Article Info form.
- If “Yes” is selected, please complete the entire form for each participant.

**Additional Notes**

- Refer to the operational definitions below for guidance on answering questions and interpreting responses.
- If you have any questions while completing the forms, please don’t hesitate to reach out to me for clarification.

Thank you again for your time and effort in coding these FCT/mand training articles!

## Appendix B

### Coding Operational Definitions

FCT/Mand Training-Article Info Forms		
Section 1: Article Information		
Question:	Response	Operational Definition
Your Name	Input your name	
Primary or Reliability Data	Primary	Primary data collector, as indicated by PI
	Reliability	Interrater agreement data collector, as indicated by PI
First author’s last name and first initial	Input the author’s last name and initial of first name separated by a comma	
Year of Publication	Select the year that the article was published in the journal	If the article has been published online and in print, input the in-print year; If the article is only published as an online version, input the online publication year
Article Title	Write the article title in its entirety. Title case is not necessary	
Was the article published in or after 2014?	Yes	The article publication year (either in print or online) is 2014–2024; if the article was available online before 2014 but available in print in 2014 or later, select “yes”
	No (exclude)	The article publication year is 2013 or earlier
Was the article published in a peer-reviewed journal?	Yes	Non-peer reviewed journals were ruled out during search, so any study published in a journal will be from a peer-reviewed journal
	No (exclude)	The article was not published in a journal. Examples include invited talks, unpublished theses or dissertations
Was the article written in a language other than English?	Yes (exclude)	
	No	
Did the article describe the teaching procedures (e.g., prompting procedure, reinforcement schedule) of at least one FCR/Mand?	Yes	
	No (exclude)	
Does the article describe a single-subject study that manipulated at least one independent variable and measured at least one dependent variable (i.e., not a review or discussion paper)?	Yes	The authors manipulated an independent variable for one or more participants and data are reported on dependent variable(s) using a single-subject research design.
	No (exclude)	The study incorporated a group design (e.g., experimental group and control group) or was a non-experimental study. Examples of non-experimental studies include descriptive studies (i.e., collection of observations without manipulating an IV), review papers, or theoretical papers.
Number of participants	Participants are defined as the number of individuals who received either mand training or FCT. Participants do not include caregivers, teachers, or staff members who were trained to implement FCT or mand training.	
Did any of your responses indicate to exclude this article?	Yes	
	No	
Section 2: General Procedures		
Question:	Response	Operational Definition
Was challenging behavior reduction targeted in the study?	Yes	The article described a response topography that was targeted for reduction with the use of a functional communication response. Examples of behaviors include aggression, pica, disruptive behavior.
	No	
	Unsure	
If yes (or unsure, if applicable), what components of functional behavior assessment(s) were used? If challenging behavior was not targeted, skip this question and move to the next question. (Select all that apply)	Indirect (e.g., interview)	Asking questions about the behavior of interest without directly observing the behavior. Examples include the FAST or Open-Ended Interview.
	Direct Observation (e.g., ABC Data, Structured Observation)	Implementers directly observed the behavior of interest by did not systematically manipulate antecedent and consequent events. Examples include ABC data collection, structured observations, or scatterplots.
	Experimental Analysis (e.g., FA, IISCA)	Implementers systematically manipulate antecedent and/or consequent events. Will include at least one test condition and a control condition.
	N/A	Select if challenging behavior reduction was not targeted in the study
	Procedure Varied by Participant	Select if one or more of the participants had differing procedures.
Method for selecting FCR/Mand topography (Select all that apply)	Mand Topography Assessment (Experimental)	Experimental assessment used to systematically identify preference for or efficiency of FCR/Mand topographies
	Caregiver Interview	Can include a formal or informal interview with a caregiver or other relevant stakeholder. Only score if article specifically states that the caregiver selected or informed the mand topography
	Therapist/Researcher Selection	Score if the article specifically states that the therapist or researcher selected the FCR/Mand topography
	Not Specified	
	Procedure Varied by Participant	Select if one or more of the participants had differing procedures.
Mastery criteria for FCR/Mand acquisition (Select all that apply)	Reduction in Challenging Behavior (e.g., 80% decrease from baseline, less than 20% of trials)	A reduction in challenging behavior was required to determine mastery of the FCR/Mand. This option can be selected in combination with other options.
	Increase in Independent FCR/Mand (e.g., above 80% correct independent)	Mastery is determined by the number of correct or independent FCRs/Mands. Does not include other types of responses such as indicating responses or correspondence checks. This option can be selected in combination with other options.
	Single-Response Criteria (e.g., correct response in cold probe, correct first-trial responding)	Mastery is determined by a single response, typically defined as the first opportunity to respond following a certain time frame. Examples include cold probes (i.e., first trial in a given day) or correct first-trial responding (i.e., first trial in a trial block)
	Repeated performance over a set number of trials/sessions	Select if the mastery criteria requires repeated performance of the response over a specified number of trials or sessions. Example: 100% correct responding over three consecutive sessions
	Suppressed responding during AO trials (e.g., less than 33% of sessions; differentiated responding)	Select if the authors used AO trials during teaching and included a requirement for differentiated responding between EO and AO trials. The authors will indicate that mastery criteria required zero or a low amount of responses during AO trials.
	Not Specified/Unknown	
	Procedure Varied by Participant	Select if one or more of the participants had differing procedures.
Number of FCRs/Mands targeted from throughout the study	1	Select the total number of FCRs/Mands that were targeted throughout the study. Not all FCRs/Mands need to be taught simultaneously (e.g., an array of 3 FCRs/Mands taught across phases or introduced sequentially); score the total number of distinct FCRs/Mands targeted for the participant. Count each FCR/Mand as distinct if it differs by: - Function (i.e., request for a different reinforcer), - Modality (e.g., vocal vs. card exchange), - Mand frame or phrasing, only if explicitly targeted as part of the intervention. Mand frame is considered explicitly targeted only if one or more of the following applies: 1. It was a stated goal or phase of the teaching procedure (e.g., shaping “I want X” from “X”). 2. It was prompted or reinforced differentially (e.g., full phrase required for reinforcement). 3. A lag schedule or other strategy was used to promote phrasing variability. 4. The study reported phrasing variation as a DV. 5. The participant was required to emit distinct phrases to access the reinforcer (e.g., taught multiple vocal frames or sentence structures). Do not count spontaneous variation in phrasing or frame unless one of the above criteria is met.
	2	
	3	
	4+	
	Not Specified	Score if the article did not describe the number of responses targeted from the onset of procedures and did not describe a teaching procedure that allowed for free-operant responding (e.g., discrete trial instruction)
	Procedure Varied by Participant	Select if one or more of the participants had differing procedures.
If distractors were used, how many distractors were used at the onset of the procedures?	0	Only score if selection-based responses (e.g., card exchange, card touch, PECS) were targeted and distractors (i.e., non-targeted responses or stimuli). Select the number of additional cards (i.e., all cards not targeted as a FCR/Mand) used as distractors at the onset of teaching. Distractors can be blank cards, cards displaying neutral or non-preferred items, or non-targeted preferred items. Select “0” if distractors were used during procedures but not during the onset of training (e.g., one target response w/o distractors at onset and eventually added blank distractors to the array).
	1	
	2	
	3+	
	N/A—Distractors Were Not Used	Select if selection-based responses were not targeted (e.g., vocal, signs) or if distractor cards were not used despite the use of a selection-based topography
	Not Specified	Select if the article indicates that distractors were used, but did not indicated the number of distractors used at the onset of teaching
	Procedure Varied by Participant	Select if one or more of the participants had differing procedures.
If distractors were used, what type of distractor cards were used	Blank cards (any color)	Distractor cards did not have any pictures, symbols, words, etc. and were blank. The color of the card (e.g., white distractor card, black distractor card) did not matter
	Low-preferred or neutral items	Distractor cards included pictures of other items, but the corresponding items were low- or neutral-preferred. Experimenters may have described a systematic test to identify value (e.g., preference assessment) or arbitrarily selected the items
	N/A—Distractors Were Not Used	Select if selection-based responses were not targeted (e.g., vocal, signs) or if distractor cards were not used despite the use of a selection-based topography
	Not Specified	Select if the article indicates that distractors were used, but did not describe the distractor
	Procedure Varied by Participant	Select if one or more of the participants had differing procedures.
Method to Increase FCR/Mand Repertoire	Teach each FCR/Mand in isolation as a simple discrimination (no conditional discrimination targeted)	Select if authors only taught a series of simple discriminations and never included all FCRs/Mands in a conditional discrimination. If the topography was vocal, select this option if the authors only arranged EOs for one FCR/Mand at a time.
	Teach each FCR/Mand in isolation and then combine all FCRs/Mands	Select if authors only taught each FCR/Mand in isolation as a simple discrimination and then combined all FCRs/Mands. For example, teaching juice, milk, and water individually and then combining all three into an array. This excludes teaching FCRs/Mands in isolation and then systematically introducing FCRs/Mands into an array.
	Teach one FCR/mand in isolation and then systematically add new FCRs/mands (conditional discrimination)	Select if authors initially taught one FCR/Mand in isolation and then systematically added one or more into the array/discrimination.
	Target all FCRs/Mands from the onset of training	All FCRs/Mands were targeted at the onset of training as a conditional discrimination. Can include two or more requests. Will also include teaching two or more requests as a conditional discrimination and then teaching additional requests later.
	N/A—only one FCR/Mand was taught	
	Not Specified	
	Procedure Varied by Participant	Select if one or more of the participants had differing procedures.
Method to Contrive EOs (Select all that apply)	Incidental Teaching	The EO was captured or contrived within the natural environment, often by placing a reinforcer in view but out of reach or interrupting access to an item the participant is currently engaging with. Mand opportunities are created by brief environmental manipulations or responses to participant behavior (e.g., reaching, pointing) rather than structured task demands. Examples: toy on high shelf, brief removal of preferred item, hiding materials mid-play.
	Interrupted Chains Procedure	The EO was contrived by interrupting a well-established behavior chain in which one or more components are missing, broken, or inoperable. The interruption creates a conditioned EO (CEO-T) by making a specific item or action necessary to complete the chain and access a terminal reinforcer. Examples: spoon missing from soup routine, puzzle piece removed, toy without batteries.
	Programmed Restriction of Reinforcers	The EO was contrived by systematically withholding access to a known reinforcer for a programmed duration (e.g., minutes, hours, days), regardless of context. The mand is evoked without any direct cues for item availability and occurs under deprivation rather than task interruption. Examples: withholding tablet or snack before session, storing preferred items out of sight until FCR occurs
	Not Specified	
	Procedure Varied by Participant	Select if one or more of the participants had differing procedures.
Prompting Type (Select all that apply)	Echoic Prompt	Therapist engages in an antecedent vocal-verbal response that shares formal similarity (i.e., both spoken) and point-to-point correspondence. Also include echoic prompts that are partial echoic prompts (i.e., antecedent response was only part of the targeted response).
	Vocal Prompt	Therapist provides an antecedent vocal-verbal response that shares formal similarity but does not include point-to-point correspondence. Examples include reminding the participant of the contingencies (“if you want something, you can touch the card”) or providing vocal statements following an indicating response (“what do you want?”)
	Model Prompt	Therapist demonstrates how to complete the target response or uses their body to gesture towards the completion of a response. This is a “show” prompt, where the therapist does not use vocal behavior or physical guidance to have the participant complete the instruction
	Physical Guidance	Therapist prompts the participant using any level of physical guidance (e.g., hand-over-hand, graduated guidance). Physical prompting should be scored if the therapist touches the participant in any way to prompt them towards completing the instruction.
	Not Specified	
	Procedure Varied by Participant	Select if one or more of the participants had differing procedures.
Prompt Fading Procedure (Select all that apply)	Most-to-Least Prompting	Therapists started with the most intrusive prompt (e.g., physical guidance, full vocal model) and then systematically decreases the level of prompting. Note: most-to-least prompting only changes the type of prompt used, not the time until a prompt. If a time delay is included, select Time Delay
	Least-to-Most Prompting	Therapists started with a lesser intrusive prompt and then systematically increased the level of prompting following errors or no-responses. Sometimes referred to as “three-step prompting” or “three-step guided compliance”
	Time Delay (e.g., fixed-time, progressive-time)	Teaching starts with the simultaneous presentation of the start of a trial (e.g., removal of item, blocked response) and the prompt. A delay is then inserted to increase the time between the start of the trial and prompt. Time delay can either be progressive (i.e., prompt delay continues to increase) or constant (i.e., once the delay is inserted, the delay remains the same)
	Stimulus Fading	Some feature of the prompt (e.g., salience) is systematically reduced to transfer stimulus control from the prompting stimulus to naturally occurring discriminative stimuli
	Not Specified	
	Procedure Varied by Participant	Select if one or more of the participants had differing procedures.
Prompt Delay or Duration of EO (Select all that apply)	0 s	A prompt delay is the amount of time between the discriminative stimulus and the prompt. For example, a preferred item is removed and the therapist waits 10 s for the participant to emit a response. Select all prompt delay durations that were used in the study. Select only the prompt delay to the teaching of the FCR or mand and not to other responses.
	1–5 s	
	6–10 s	
	11–30 s	
	Greater than 30 s	
	Not Specified	
	Procedure Varied by Participant	Select if one or more of the participants had differing procedures.
Response Specifying Reinforcer Provided (i.e., what did the participant mand for?)	Functional Reinforcer (i.e., same reinforcer that maintains challenging behavior)	Select if challenging behavior reduction was targeted and the reinforcer for the FCR was the same reinforcer that maintained challenging behavior, as indicated by the FBA
	Non-Functional Reinforcer (Challenging behavior still targeted but FCR was for a reinforcer that maintains target behavior)	Select if challenging behavior reduction was targeted, but the reinforcer for the FCR was different from the reinforcer maintained challenging behavior, as indicated by the FBA. If both a functional and nonfunctional reinforcer was provided for FCRs, select both options.
	Toys	Select the appropriate reinforcer type only if challenging behavior was not targeted for reduction. Select only the reinforcer that was specified in the mand (i.e., mand-specifying reinforcer) and not and additional reinforcers provided following the mand.
	Food-Based	
	Attention	
	Tokens	
	Information	
	Access to Routine	
	Access to Activity	
	Not Specified	
	Procedure Varied by Participant	Select if one or more of the participants had differing procedures.
What Additional Consequences Were Provided Along with the EO-Related Reinforcer	See above for options and operational definitions	
Parameter of Reinforcement (Select all that apply)	Time-based	Select if authors described a time-based parameter of reinforcement, such as 30-s access to an item or remainder of trial. Selection of time-based indicates that the researcher takes away, or is able to take away, the reinforcers after a set duration of time. If the authors describe reinforcement as “brief access to (e.g., 3–5 s)”, select a time-based parameter.
	Discrete Unit	Select if the reinforcer was a discrete unit of reinforcement instead of being based on an amount of time. Examples: one skittle, information, a statement on praise). If the authors describe reinforcement as “brief access to (e.g., 3–5 s)”, select a time-based parameter.
	Not Specified	
	Procedure Varied by Participant	Select if one or more of the participants had differing procedures.
Was a method to check correspondence (e.g., correspondence check, indicating response, measure of consumption) between the EO and the mand included?	Yes	Select if the authors included a method to check correspondence between the EO and the mand. Methods can include an indicating response (therapists waited until participant showed interest in the reinforcer), correspondence check (therapist had participant select the item from an array following the mand to ensure the mand and selected item matched), or measure of consumption (authors measured if the participant consumed the reinforcer)
	No	No method to check correspondence was used.
	Procedure Varied by Participant	Select if one or more of the participants had differing procedures.
Session Arrangement	Duration-Based Sessions (e.g., 10-min session)	Data were collected during sessions that were based on a session duration. The session lengths could vary, but were not defined by the number trials or requests during the session
	Response-Based Sessions (e.g., 10 trials)	Sessions were defined by the number of opportunities to emit a response (i.e., trials) and not based on a duration of session.
	Not Specified	
	Procedure Varied by Participant	Select if one or more of the participants had differing procedures.
FCT/Mand Training-Participant Info Forms		
Section 2: Participant Information		
Question:	Response	Operational Definition
Participant Name		
Age of Participant (in years) If both years and months were reported, round the months to the nearest year (e.g., 11 years and 3 months to 11 years, 5 years and 10 months to 6 years)		
Participant Diagnoses (select all that apply)	Autism Spectrum Disorder (aka Autistic, Autism, ASD, Autistic Disorder)	
	Developmental Disability (e.g., Down Syndrome, Global Developmental Delay)	
	Attention-Deficit Hyperactivity Disorder (ADHD [other names include ADD])	
	Dementia or other Degenerative Disorder	
	No Diagnoses	
	Not Specified	
Assessment(s) of Language/Communication	Peabody Picture Vocabulary Test (PPVT)	
	Verbal Behavior Milestones Assessment and Placement Program (VB-MAPP)	
	Early Echoic Skills Assessment (EESA)	
	VB-MAPP Intraverbal Assessment Subtest	
	Assessment of Basic Language and Learning Skills (ABLLS or ABLLS-R)	
	Vineland Adaptive Behavior Scale	
	Expressive One Word Picture Vocabulary Test (EOWPVT)	
	Expressive Vocabulary Test (EVT)	
	Preschool Language Scale (PLS)	
	Information on Number of Mands	
	Information on Sentence Length (e.g., 1-to-2 word sentences)	
	Information from IEP	
	None Reported	
What were the reported scores? Please provide the score exactly as it was reported in the article. You can use a direct quote to report scores. If multiple assessments or scores were reported, separate them by using a semicolon (If no information was provided on language, type in N/A)		
FCR/Mand Topography	Vocal	
	Picture Communication (e.g., picture exchange, card touch)	
	Speech Output Device (e.g., tablet with Dynavox, Bigmac Button)	
	Sign (i.e., not ASL; e.g., modified signs)	
	ASL	
	Pointing/Gestures	
	Not Specified	
Therapy Setting	Clinic (outpatient)	
	School	
	Home	
	Hospital (inpatient)	
	Institution (Group home, Nursing home)	
	Vocational Program	
	Not Specified	
Section 3: Participant Challenging Behavior Information		
Question:	Response	Operational Definition
Topography(ies) of Behavior Targeted with Intervention	Aggression	
	Self-injury	
	Disruptions/Property Destruction	
	Negative Vocalizations	
	Stereotypic Behavior (Vocal or Motor)	
	Tantrums	
	Inappropriate Mealtime Behavior	
	Elopement	
	Noncompliance	
	Pica	
	Enuresis or Encopresis	
	Rumination and/or Operant Vomiting	
	Dangerous Behavior (e.g., setting fires, climbing)	
Function(s) of Behavior Targeted with Intervention	Attention	
	Tangible	
	Escape	
	Automatic	
	Synthesized	
	Social Control (also called mand compliance, adult compliance with mands, request compliance, compliance with requests, adult-directed play, escape to child-directed play)	
	Access to Ritualistic Behavior (e.g., arranging and ordering)	
	Not Specified	
Functional Behavior Assessment Type	Indirect (e.g., interview)	Asking questions about the behavior of interest without directly observing the behavior. Examples include the FAST or Open-Ended Interview.
	Direct Observation (e.g., ABC Data, Structured Observation)	Implementers directly observed the behavior of interest by did not systematically manipulate antecedent and consequent events. Examples include ABC data collection, structured observations, or scatterplots.
	Experimental Analysis (e.g., FA, IISCA)	Implementers systematically manipulate antecedent and/or consequent events. Will include at least one test condition and a control condition.
	Not Specified	
	None	
Section 4: Participant FCT/Mand Training Variations		
Question:	Response	Operational Definition
Did you select “Procedure Varied by Participant” for any of the responses in the FCT/Mand Training-Article Info survey?	Yes	
	No	
